# Photoheterotroph improved the growth and nutrient levels of *Chlorella vulgaris* and the related molecular mechanism

**DOI:** 10.1007/s00253-024-13090-w

**Published:** 2024-03-20

**Authors:** Xianmei Long, Cancan Zhang, Qian Yang, Xiaorui Zhang, Wangwang Chen, Xiaofang Zhu, Qing Xu, Qingsong Tan

**Affiliations:** 1 National Demonstration Center for Experimental Aquaculture Education, College of Fisheries, Huazhong Agricultural University, Wuhan, 430070 China; 2 Key Laboratory of Freshwater Animal Breeding, Ministry of Agriculture, Wuhan, 430070 China; 3 Engineering Research Center of Green Development for Conventional Aquatic Biological Industry in the Yangtze River Economic Belt, Ministry of Education, Wuhan, 430070 China; 4 Hubei Vocational College of Bio-Technology, Wuhan, 430070 China

**Keywords:** *Chlorella vulgaris*, Trophic mode, Growth characteristics, Nutrients, Transcriptome analysis

## Abstract

**Abstract:**

Microalgae are rich in fatty acids, proteins, and other nutrients, which have gained the general attention of researchers all over the world. For the development of *Chlorella vulgaris* in food and feed industry, this study was conducted to investigate the differences in *C. vulgaris*’ growth and nutritional components under different culture conditions (autotrophic, heterotrophic, photoheterotrophic) and the internal factors through cell counting in combination with transcriptome and nutrient analyses. The results showed that, under the photoheterotrophic condition, *Chlorella*’s growth and the contents of lipid and protein were significantly higher than that under the heterotrophic condition, and the moisture content was lower than that under the heterotrophic condition. The saturated fatty acid content under the photoheterotrophic condition was the lowest, while the polyunsaturated fatty acid content was significantly higher than those under the other two conditions. There were 46,583 differentially expressed genes (DEGs), including 33,039 up-regulated DEGs (70.93%) and 13,544 down-regulated DEGs (29.07%), under the photoheterotrophic condition in comparison with the autotrophic condition. The fold change between the two conditions of samples of up-regulated genes was higher than that of the down-regulated genes. The KEGG enrichment showed that the up-regulated DEGs in the photoheterotrophic condition were significantly enriched in 5 pathways, including protein processing in endoplasmic reticulum pathway, photosynthesis pathway, photosynthesis-antenna protein pathway, endocytosis pathway, and phosphonate and phosphinate metabolism pathway. DEGs related to fatty acid metabolic pathways were significantly enriched in the fatty acid biosynthesis pathway and the biosynthesis of unsaturated fatty acid pathway. The qPCR analysis showed that the expression pattern of the selected genes was consistent with that of transcriptome analysis. The results of this study lay a theoretical foundation for the large-scale production of *Chlorella* and its application in food, feed, and biodiesel.

**Key points:**

*• Nutrient levels under photoheterotrophic condition were higher than other conditions.*

*• Six important pathways were discovered that affect changes in nutritional composition.*

*• Explored genes encode important enzymes in the differential metabolic pathways.*

**Supplementary Information:**

The online version contains supplementary material available at 10.1007/s00253-024-13090-w.

## Introduction

*Chlorella* is a genus of microalgae that belongs to the family *Chlorellaceae*, order *Chlorococcale*, class *Chlorophyceae*, division *Chlorophyta*, and consists of mostly green algae living in freshwater and marine environments. *Chlorella* species are well known for the richness in protein and lipid, which have been widely studied for their potential application in food and feed industry, biofuel production, and wastewater treatment (Aly et al. 2023; Choong et al. 2020). The protein content of *Chlorella* could be at a very high level of 60% in dry weight, which is higher than that of soybean powder. Therefore, microalgae with high protein have become an ideal substitute for fish meals in aquatic feed (Bito et al. 2020; Mišurcová et al. 2014). Especially, the cell wall of *Chlorella* is mainly composed of proteins, which are rich in leucine, arginine, and lysine lacking in the cereal proteins (Hemaiswarya et al. 2011). Therefore, *Chlorella* can be used in compound feed to achieve the purpose of “amino acid complementation” so as to solve the problem of the low nutritional value of plant protein (Leong et al. 2020; Mišurcová et al. 2014). Moreover, the important unsaturated fatty acids contained in *Chlorella* are EPA (eicosapentaenoic acid) and DHA (docosahexaenoic acid), which are necessary and cannot be synthesized by aquaculture animals themselves (Barta et al. 2021).

The trophic modes of *Chlorella* have been investigated for the development of its potential in the biodiesel production (Choong et al. 2020). Under the autotrophic condition, *Chlorella* can use inorganic carbon sources (such as CO_2_) to convert light energy into its available chemical energy; however, the particularly low biomass productivity and lipid productivity of *Chlorella* under this condition has also become the bottleneck of *Chlorella* in the large-scale production of biodiesel (Chisti 2007). In order for the cost-effective algae oil production, heterotrophic condition, eliminating the requirement of light and using organic carbon sources (such as glucose and organic acids), was developed to significantly improve the cell density and productivity (Chen 1996; Liang et al. 2009). However, it is expensive and easy to pollute the algae. Integrating the advantages of phototrophic and heterotrophic condition, *Chlorella* can be cultured under the photoheterotrophic condition through simultaneously utilizing inorganic carbon sources for autotrophic and organic carbon sources for heterotrophy (Barta et al. 2021; Liang et al. 2009). This culture model shows higher lipid and biomass productivity, lower photoinhibition, and less pollution to the algae (Ananthi et al. 2021).

*Chlorella vulgaris*, which widely exists in natural water bodies, is an important resource microalga belonging to the *Chlorella* genus, and also is the first artificially cultivated microalgae (Mitra et al. 2012). *C. vulgaris* is rich in protein (about 42–58% of dry weight), unsaturated fatty acids (linoleic acid, linolenic acid, EPA, DHA, etc.), carotenoids, astaxanthin, a variety of vitamins, and active metabolites (Safi et al. 2014), which show its high nutritional value and the potential in improving immunity for human and animals. Moreover, the high photosynthetic ability and the rapid growth under autotrophic, photoheterotrophic, and heterotrophic conditions of *C. vulgaris* made it one of the first microalgae considered for large-scale cultivation and commercial production (Borowitzka 2018).

However, the differences in the growth and the nutrient level among the 3 culture conditions lack a systematic evaluation. To understand the differences in *Chlorella*’s nutritional components under different culture modes and the internal factors that cause this difference, the current study was conducted through the *C. vulgaris* culture under the three culture modes followed by the cell counting, nutrient analyses, and the transcriptome analysis. The results of this can lay a theoretical foundation for the large-scale production of *Chlorella* and its application in food, feed, and biodiesel.

## Materials and methods

### Microalgae and culture conditions

*C. vulgaris* FACHB-2723 (obtained from Freshwater Algae Culture Collection of the Institute of Hydrobiology, Chinese Academy of Science, Wuhan, China) was used in this study. The ingredients of BG11 medium used in the experiments are listed in Supplemental Tables S1 and S2. The autotrophic condition was run using 100 mL of medium BG11 in 250-mL flasks illuminated from one side with bubbling of sterilized air containing 5% CO_2_ (5% CO_2_-air) at 25 °C. The light source used was 40-W daylight fluorescent lamps at 10 kilolux at the surface of medium. The heterotrophic condition was performed using 100 mL of medium BG11 containing 10 g/L of glucose solution and 50 µg/mL of ampicillin solution (Biofroxx, Einhausen, Germany) in each 250-mL cotton plugged Erlenmeyer flask, on a thermostatic shaker (216 rpm) at 25 °C in the dark. For the photoheterotrophic condition, the illumination and culture process were the same as in the autotrophic condition except that the culture medium BG11 contained 10 g/L of glucose solution and 50 µg/mL of ampicillin solution. Algae in logarithmic growth phase were seeded at a density of 5 ~ 10 × 10^5^ cells/mL into a 250-mL conical flask containing 100-mL culture medium, and then transferred to the corresponding culture conditions. During the culture process, the culture medium was changed every 3 days. All of the experiments were repeated at least 3 times to assure three separate flasks (replicates) analyzed at the same time.

### Evaluation of growth performance

*Chlorella* in the logarithmic growth phase was inoculated at the same density (1.0 × 10^6^ individuals/mL) and cultured for 15 days under the autotrophic condition, heterotrophic condition, or photoheterotrophic condition. The cells were harvested at 24 h intervals until 15 days. The harvested cells were counted by a cell counter (Countstar BioMed, Shanghai, China) in triplicates for each sampling time point, and the average cell count was used to plot the growth curve.

### Sample collection

According to the growth curve of *C. vulgaris* mentioned above, at the 7th day of *Chlorella* culture, the biomass obtained was harvested by centrifugation at 1700 × *g* for 10 min, then fast frozen in liquid nitrogen, and stored at − 80 °C for further analyses on the nutrient contents and transcriptome. Based on the growth status of the algae, only *Chlorella* samples from the autotrophic condition and the photoheterotrophic condition were tested in the transcriptome analysis.

### Determination of moisture and protein contents

The moisture content in the *Chlorella* samples was determined by vacuum freeze-drying method. After dehydration, the total protein content was analyzed using the Kjeldahl method with a nitrogen-to-protein conversion factor of 6.25, as described by the national standard of the People’s Republic of China (GB/T5009.5–2003).

### Lipid and fatty acid profile analyses

Total lipid of the frozen-dried *Chlorella* meal was determined using the chloroform–methanol (1: 2, v/v) extraction method (de Jesus et al. 2018).

After determining the lipid content, the dried residue was methylated with boron trifluoride (BF_3_) in methanol, and then the methyl esterified samples were analyzed by gas chromatography (Agilent 6890, Santa Clara, CA, USA) with an OmegawaxTM320 column (Agilent 6890, Santa Clara, CA, USA). Chromatography was carried out as described in our recent literature (Zhou et al. 2023). Individual fatty acid methyl ester (FAME) peaks were identified according to their retention times in comparison to the retention times of known FAMEs in a FAME standard (Nu-Chek Prep. Inc, Elysian, MN, USA) containing 40 FAMEs. Identified fatty acids were presented as an area percentage of the total fatty acid (Ördög et al. 2013).

### Amino acid analysis

For amino acid analysis, dried algae were ground and 0.1 g samples were hydrolyzed with 10 mL hydrochloric acid (6 M) at 110 °C in microreaction vials under nitrogen for 24 h. Following hydrolyzation and derivatization, the sample was measured by a fully automated amino acid analyzer (Hitachi L-8800, Hitachi Ltd., Tokyo, Japan) for the detection of 16 amino acids, including aspartic acid, threonine, serine, glutamic acid, glycine, alanine, valine, methionine, isoleucine, leucine, tyrosine, phenylalanine, histidine, lysine, ammonium chloride, and arginine.

### Transcriptome data analysis

Total RNA was extracted from the cells using TRIzol® Reagent according the manufacturer’s instructions (Invitrogen, Carlsbad, CA, USA) and genomic DNA was removed using DNase I (TaKaRa, Tokyo, Japan). RNA degradation and contamination were monitored on 1% agarose gels. Then the integrity and purity of the total RNA quality were determined by 2100 Bioanalyser (Agilent Technologies, Santa Clara, CA, USA) and the RNA content was quantified using the ND-2000 (NanoDrop Technologies, Waltham, MA, USA). Only a high-quality RNA sample (OD260/280 = 1.8 ~ 2.2, OD260/230 ≥ 2.0, RIN ≥ 8.0, 28S: 18S ≥ 1.0, > 1 μg) was used to construct sequencing library. The transcriptome library was prepared following a TruSeq TM RNA sample preparation Kit (Illumina, San Diego, CA, USA) using 1 g of total RNA. Shortly, messenger RNA was isolated according to the poly-A selection method by oligo (dT) beads and then fragmented by fragmentation buffer firstly. Secondly double-stranded cDNA was synthesized using a SuperScript double-stranded cDNA synthesis kit (Invitrogen, Carlsbad, CA, USA) with random hexamer primers (Illumina, Santa, Clara, CA, USA). Then the synthesized cDNA was subjected to end-repair, phosphorylation, and ‘A’ base addition according to Illumina’s library construction protocol. Libraries were size selected for cDNA target fragments of 300 bp on 2% Low-Range Ultra Agarose followed by PCR amplified using Phusion DNA polymerase (NEB, Ipswich, MA, USA) for 15 PCR cycles. After quantified by Qubit 4.0 fluorometer (Thermo Scientific, Waltham, MA, USA), paired-end RNA-seq sequencing library was sequenced with the Illumina NovaSeq 6000 sequencer (2 × 150-bp read length; Illumina, San Diego, CA, USA) by Shanghai Majorbio Bio-pharm Biotechnology Co., Ltd. (Shanghai, China).

The software fastp (https://github.com/OpenGene/fastp) was used for the adapter trimming and quality control of the raw paired-end reads with default parameters. Briefly, clean reads obtained by removing adapter reads and low-quality reads (quality score < 20) were used to do de-novo assembly with Trinity (http://trinityrnaseq.sourceforge.net/). Then the assembled transcripts were assessed and optimized with BUSCO (Benchmarking Universal Single-Copy Orthologs, http://busco.ezlab.org), TransRate (http://hibberdlab.com/transrate/), and CD-HIT (http://weizhongli-lab.org/cd-hit). All the assembled transcripts were searched against the NCBI protein non-redundant database (NR, ftp://ftp.ncbi.nlm.nih.gov/blast/db/), Swiss-Prot (http://web.expasy.org/docs/swiss-prot_guideline.html), Pfam (http://pfam.xfam.org/), Clusters of Orthologous Groups of proteins (COG, http://www.ncbi.nlm.nih.gov/COG/), GO (Gene Ontology, http://www.geneontologo.org), and KEGG (Kyoto Encyclopedia of Genes and Genomes, http://www.genome.jp/keeg/) databases using BLASTX to identify the proteins that had the highest sequence similarity with the given transcripts to retrieve their function annotations. A typical cut-off *E*-value < 1.0 × 10^−5^ was set.

The differentially expressed genes (DEGs) were obtained by differential analysis software DESeq2 (http://bioconductor.org/packages/stats/bioc/DESeq2/) according to the standards of *p*-adjust < 0.05 and | log_2_ (fold change) |≥ 1.5, and the functional enrichment of the DEGs was analyzed by Gene Ontology (GO, http://www.geneontology.org/) and Kyoto Encyclopedia of Genes and Genomes (KEGG, http://www.genome.jp/kegg/).

### Quantitative real-time PCR validation

Samples of algal cells grown under the photoheterotrophic and the autotrophic conditions were harvested and were used for qPCR validation. RNA extraction and RNA quality check were conducted as previously described. The Hifair® III 1st Strand cDNA Synthesis SuperMix for qPCR (gDNA digester plus, Yeasen, Shanghai, China) was used for reverse transcription of 1 µg of total RNA according to manufacturers’ instructions. The quantitative real-time PCR (qRT-PCR) for gene expression assay was performed using Hieff® qPCR SYBR® Green Master Mix (No Rox) (Yeasen, Shanghai, China) on a quantitative thermal cycler (Light Cycler 480II, Roche, Basel. Switzerland). Specific primer pairs were designed for each target gene through Primer Premier (version 5.0) software (PREMIER Biosoft, San Francisco, CA, USA) using known sequences in the NCBI database (Supplemental Table S3). The qRT-PCR conditions were as follows: preincubation at 95 °C for 5 min, followed by 40 cycles at 95 °C for 10 s, annealing temperature (corresponding specific primer pairs) for 20 s, and 72 °C for 20 s. Melting curves were systematically monitored (temperature gradient at 0.5 °C/s from 60 to 95 °C). Using 18S as reference genes, the genes were quantified using the 2^−∆∆Ct^ value method according to Pfaffl (2001). The value of algal cell in the autotrophic condition was assigned as an arbitrary value of 1.

### Statistical analysis

All of the experiments were repeated 3 times independently (three separate flasks analyzed at the same time), and data were recorded as the mean with standard deviation (SD). The growth data and nutrient parameters were subjected to one-way ANOVA (analysis of variance) followed by the post hoc (Duncan’s multiple range test) to test the effects of trophic treatment on algal growth and nutrient contents. Differences between genes validated were identified by independent *t*-test. Statistical analyses were performed using SPSS software (version 26.0, IBM, Armonk, NY, USA), and a *p*-value < 0.05 was considered statistically significant. The threshold for the DEG identification and the KEGG pathway enrichment were set at *p*-values < 0.05 corrected by the Benjamini and Hochberg correction method.

## Results

### Growth performance of algae under different culture conditions

**Fig. 1:**
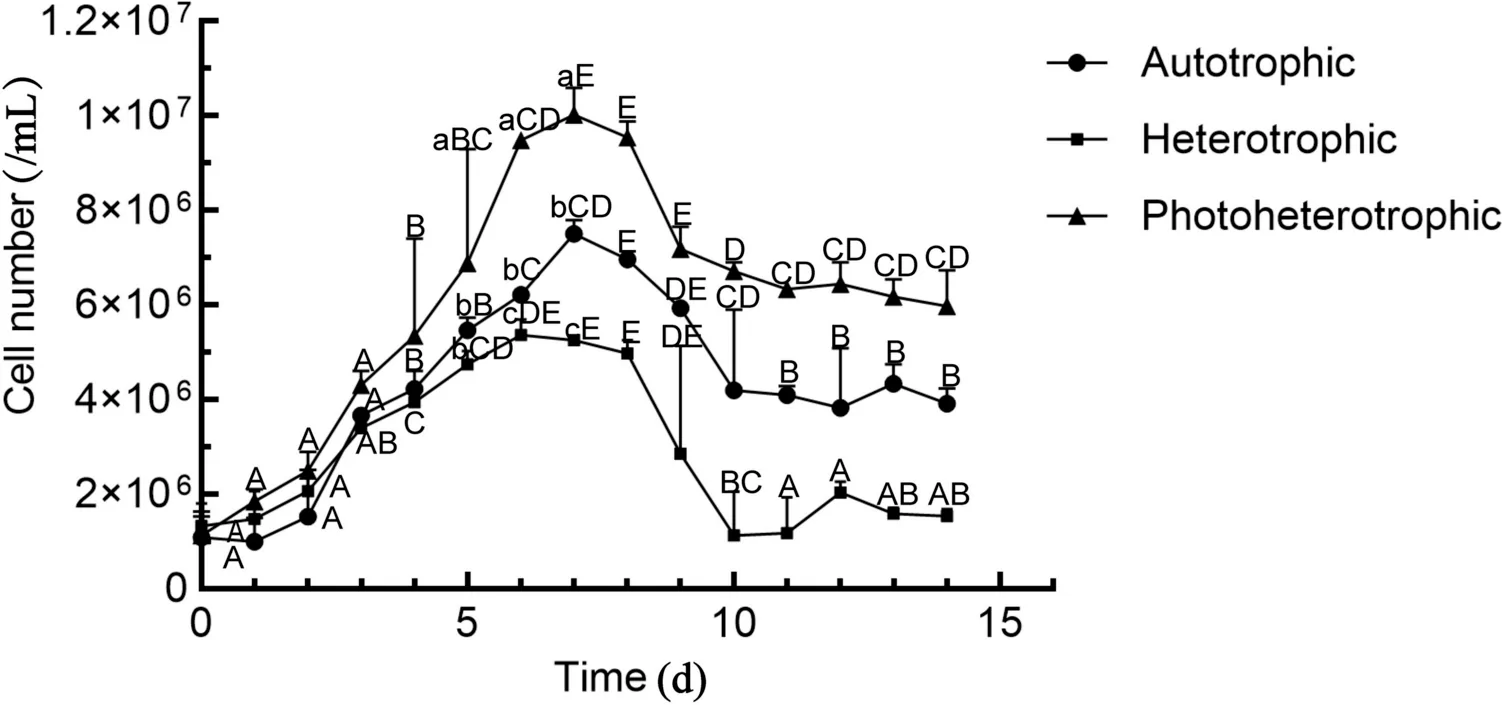
Effects of different culture conditions on the growth characteristics of *C. vulgaris*. Different capital letters indicate significant difference in the cell counting at different sampling days under the same condition, and different lowercases indicate significant difference between the different conditions at the peak day ages, day 5th, 6th, and 7th

As shown in Fig. 1, *C. vulgaris* quickly enters the rapid growth period after an adaptation period of 2 days. During the culture process, the biomass of *C. vulgaris* under the photoheterotrophic condition was the highest. Before the 3rd day, the biomass under the heterotrophic condition was higher than that under the autotrophic condition; however, after the 3rd day, the biomass under the autotrophic condition was higher than that under the heterotrophic condition. Under the autotrophic and photoheterotrophic conditions, *Chlorella* ended its logarithmic growth period after 7 days, while under the heterotrophic condition, the growth of *Chlorella* tended to decline after 6 days, and entered the plateau stage after 10 days.

### Moisture, protein, and lipid contents of algae under different culture conditions

The culture condition will affect the nutrient composition of *Chlorella*. As shown in Table 1, the protein content of algal cell was the highest under the photoheterotrophic condition, followed by the heterotrophic condition, and the lowest under the autotrophic condition. The lipid content under the photoheterotrophic condition was significantly higher than that under the heterotrophic condition, while it was not different from that under the autotrophic condition. However, the moisture content under the autotrophic condition was significantly lower than that under the heterotrophic and photoheterotrophic conditions.

**Table 1:** Lipid, protein, and moisture contents (%) of *C. vulgaris* under different culture conditions

	Autotrophic	Heterotrophic	Photoheterotrophic
Lipid	15.29 ± 0.70^ab^	12.75 ± 1.18^a^	17.39 ± 0.85^b^
Protein	23.24 ± 0.21^a^	31.19 ± 0.56^b^	42.59 ± 0.31^c^
Moisture	17.34 ± 0.79^a^	22.94 ± 1.40^b^	21.36 ± 0.53^b^

Different superscripts after the data in the same row indicate significant difference (*p*-value < 0.05). Lipid and protein contents were expressed as % in dry matter

### Fatty acid profiles of algae under different culture conditions

The effects of different culture conditions on the fatty acid composition of *C. vulgaris* are shown in Table 2. The total saturated fatty acid content under the autotrophic condition was the highest among three conditions, followed by the heterotrophic condition, and the lowest under the photoheterotrophic condition, while the total polyunsaturated fatty acid (PUFA) and n-6 PUFA contents under the photoheterotrophic condition were significantly higher than those under the autotrophic and heterotrophic conditions. In contrast, total n-3 PUFA contents under the photoheterotrophic and autotrophic conditions were significantly higher than that under the heterotrophic condition. The content of total monounsaturated fatty acids (MUFA) under the autotrophic condition was significantly lower than the other two conditions. Among them, C14:0, C20:0, C20:1n-11, C20:3n-3, C20:4n-3, C20:5n-3, and C22:1n-11 of *Chlorella* were not significantly different under different culture conditions. The contents of C18:0, C18:3n-6, C18:4n-3, C22:5n-3, and C22:6n-3 of *Chlorella* were all the highest in algal cells under the autotrophic condition, followed by cells under the heterotrophic condition, and the lowest was in cells under the photoheterotrophic condition; the contents of C14:1 and C20:4n-6 were the highest in cells under the heterotrophic condition, followed by cells under the photoheterotrophic condition, and the lowest was under the autotrophic condition. The contents of C16:0, C16:1, and C20:2n-6 were relative higher in cells under the heterotrophic condition among the three groups, but the content of C18:1n-9, C18:2n-6, and C18:3n-3 were the highest under the photoheterotrophic condition, followed by the heterotrophic condition, and the lowest was under the autotrophic condition.

**Table 2:** Fatty acid composition of *C. vulgaris* under different culture conditions (relative area percentage, %)

Fatty acid	Autotrophic	Heterotrophic	Photoheterotrophic
C14:0	0.69 ± 0.08	0.75 ± 0.02	0.51 ± 0.09
C14:1	0.69 ± 0.00^a^	1.72 ± 0.07^c^	1.12 ± 0.10^b^
C16:0	25.42 ± 0.39^a^	31.01 ± 0.75^b^	28.85 ± 0.25^b^
C16:1	2.67 ± 0.08^ab^	3.21 ± 0.68^b^	1.25 ± 0.10^a^
C18:0	35.32 ± 0.23^c^	16.68 ± 0.90^b^	6.06 ± 0.49^a^
C18:1n-9	0.02 ± 0.01^a^	15.11 ± 1.62^b^	22.37 ± 1.18^c^
C18:2n-6	10.84 ± 0.04^a^	8.67 ± 0.77^a^	14.04 ± 0.63^b^
C18:3n-6	4.16 ± 0.01^c^	0.96 ± 0.04^b^	0.76 ± 0.02^a^
C18:3n-3	10.48 ± 0.19^a^	16.32 ± 0.56^b^	21.69 ± 0.70^c^
C18:4n-3	5.29 ± 0.19^c^	1.98 ± 0.11^b^	1.21 ± 0.14^a^
C20:0	0.57 ± 0.01	0.58 ± 0.22	0.42 ± 0.08
C20:1n-11	0.22 ± 0.01	0.21 ± 0.07	0.11 ± 0.01
C20:2n-6	0.04 ± 0.01^a^	0.19 ± 0.05^b^	0.14 ± 0.01^ab^
C20:3n-3	0.11 ± 0.02	0.15 ± 0.02	0.09 ± 0.02
C20:4n-6	0.06 ± 0.01^a^	0.27 ± 0.03^c^	0.13 ± 0.01^b^
C20:4n-3	0.29 ± 0.19	0.22 ± 0.03	0.17 ± 0.06
C20:5n-3 (EPA)	0.93 ± 0.22^b^	0.14 ± 0.01^a^	0.12 ± 0.03^a^
C22:1n-11	0.16 ± 0.10	0.64 ± 0.25	0.14 ± 0.04
C22:5n-3	1.50 ± 0.03^c^	0.71 ± 0.05^b^	0.52 ± 0.02^a^
C22:6n-3 (DHA)	0.52 ± 0.01^c^	0.33 ± 0.02^b^	0.17 ± 0.04^a^
SFA	62.01 ± 1.21^c^	49.02 ± 0.36^b^	35.84 ± 0.24^a^
MUFA	3.76 ± 0.22^a^	20.89 ± 2.00^b^	24.99 ± 2.00^b^
PUFA	34.22 ± 1.00^a^	30.10 ± 2.01^a^	39.17 ± 2.08^b^
n-6PUFA	19.13 ± 1.05^a^	19.84 ± 0.91^a^	23.98 ± 1.20^b^
n-3FUFA	15.1 ± 0.06^b^	10.25 ± 1.41^a^	15.19 ± 1.18^b^

Values in each row with different superscripts are significantly different (*p*-value < 0.05). *SFA*: saturated fatty acids; *MUFA*: monounsaturated fatty acids; *PUFA*: polyunsaturated fatty acids. n-6 PUFA includes C18:2n-6, C18:3n-6, C20:2n-6, C20:4n-6; n-3 PUFA includes C18:3n-3, C18:4n-3, C20:3n-3, C20:4n-3, EPA, C22:5n-3, DHA

### Amino acid composition of algae under different culture conditions

The amino acid composition of *Chlorella* under different culture conditions are shown in Table 3. Different culture conditions significantly affected the amino acid contents of *Chlorella*. Most of the amino acids, including lysine, leucine, valine, phenylalanine, threonine, isoleucine, histidine, glycine, alanine, and glutamic acid, were the highest in the cells under the photoheterotrophic condition, followed by the heterotrophic condition, and the lowest was in the cells under the autotrophic condition. However, the tyrosine content under the heterotrophic condition was the highest, followed by that under the photoheterotrophic condition, and the lowest was in *Chlorella* under the autotrophic condition. The methionine content under the heterotrophic condition was significantly higher than in the other two groups, and the arginine content under the phototrophic condition was significantly lower than in the other two groups, while the contents of aspartic acid and serine under the photoheterotrophic condition were significantly higher than in the other two groups.

**Table 3:** Amino acid composition of *C. vulgaris* under different culture conditions (proportion of dry matter, %)

Amino acid	Autotrophic	Heterotrophic	Photoheterotrophic
EAA			
Lysine	1.02 ± 0.03^a^	1.34 ± 0.06^b^	2.27 ± 0.06^c^
Leucine	1.73 ± 0.05^a^	2.16 ± 0.09^b^	3.10 ± 0.10^c^
Valine	1.07 ± 0.03^a^	1.41 ± 0.05^b^	2.00 ± 0.07^c^
Methionine	0.34 ± 0.01^a^	0.80 ± 0.01^b^	0.36 ± 0.07^a^
Phenylalanine	1.00 ± 0.03^a^	1.24 ± 0.05^b^	1.73 ± 0.06^c^
Threonine	1.07 ± 0.03^a^	1.21 ± 0.11^b^	1.83 ± 0.06^c^
Isoleucine	0.75 ± 0.02^a^	1.00 ± 0.06^b^	1.44 ± 0.05^c^
Arginine	0.58 ± 0.02^a^	0.90 ± 0.09^b^	1.00 ± 0.06^b^
Histidine	0.24 ± 0.02^a^	0.40 ± 0.04^b^	0.64 ± 0.02^c^
NEAA			
Glycine	1.36 ± 0.04^a^	1.60 ± 0.08^b^	2.31 ± 0.07^c^
Aspartic acid	1.48 ± 0.05^a^	1.72 ± 0.11^a^	2.40 ± 0.07^b^
Serine	0.95 ± 0.03^a^	1.07 ± 0.08^a^	1.70 ± 0.05^b^
Tyrosine	0.57 ± 0.09^a^	1.65 ± 0.25^c^	1.11 ± 0.15^b^
Alanine	1.76 ± 0.05^a^	2.20 ± 0.08^b^	3.11 ± 0.10^c^
Glutamic acid	2.21 ± 0.07^a^	2.91 ± 0.14^b^	4.38 ± 0.13^c^
AA total	16.12 ± 0.50^a^	21.20 ± 0.55^b^	29.82 ± 0.89^c^

Values in each row with different superscripts are significantly different (*p*-value < 0.05). *EAA*: essential amino acid; *NEAA*: non-essential amino acids

### Raw data analysis of transcriptome

**Fig. 2:**
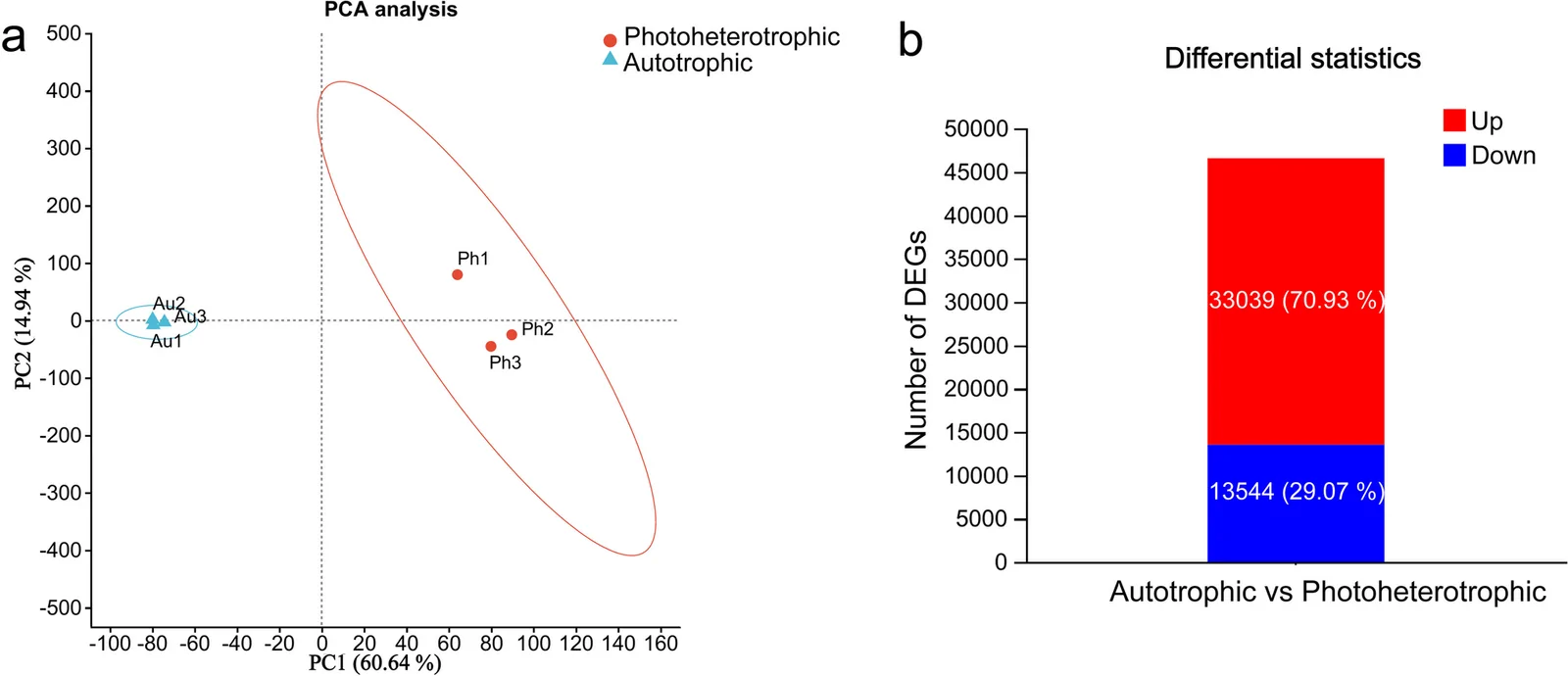
Raw data analysis of transcriptome. **a** PCA analysis between samples. **b** Analysis of expression difference (up: up-regulated genes in differential genes; down: down-regulated genes in differential genes)

Figure 2a shows that the biological duplication of the autotrophic condition is better than that of the photoheterotrophic condition, and a clear separation between samples of the autotrophic condition and the photoheterotrophic condition. Compared with the autotrophic condition, there were 33,048 up-regulated DEGs and 13,568 down-regulated DEGs under the photoheterotrophic condition (Fig. 2b).

### GO and KEGG enrichment analysis on DEGs of algae under different culture conditions

**Fig. 3:**
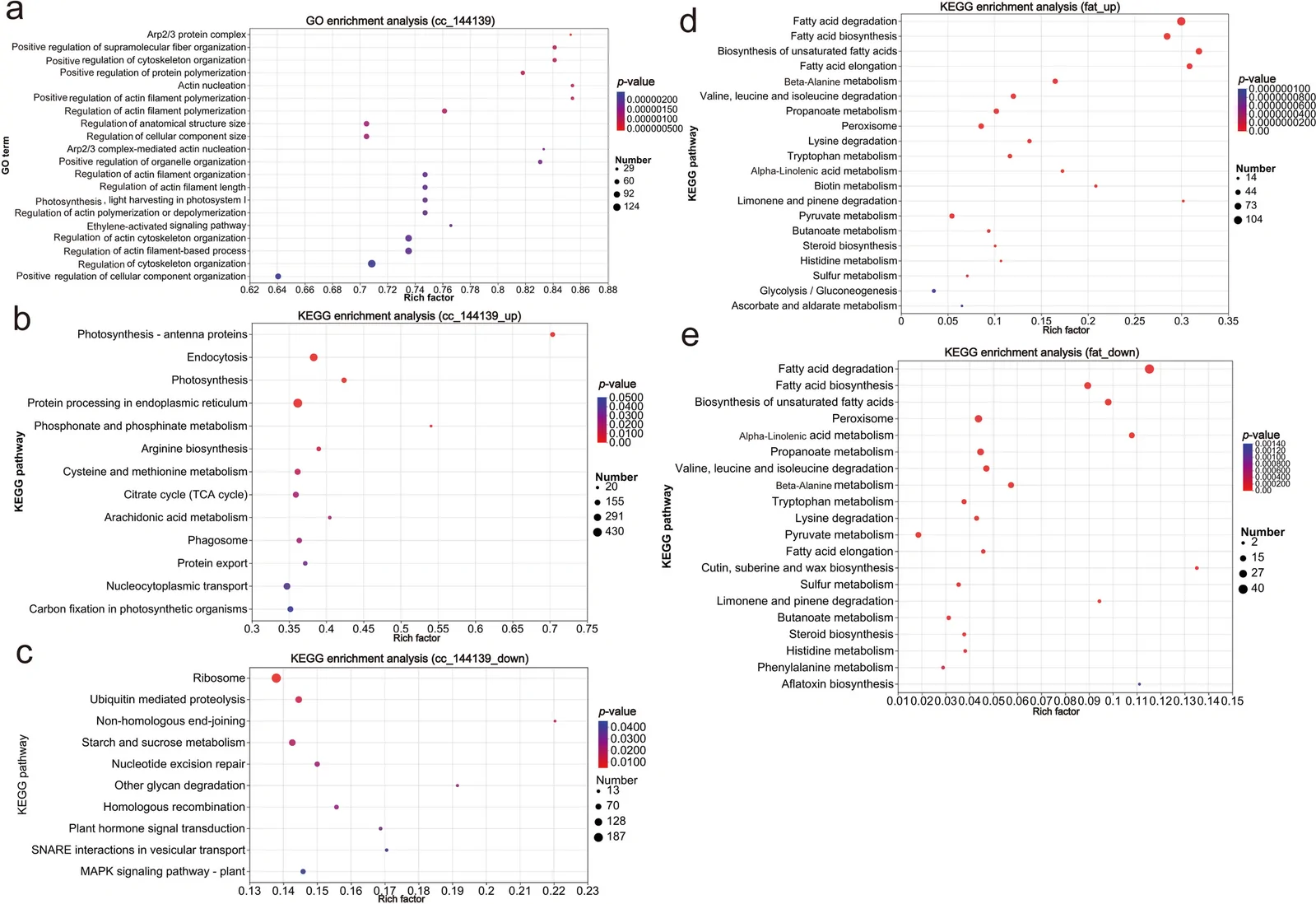
Results of GO and KEGG enrichment analysis. **a** GO enrichment analysis (*p* value-corrected < 0.05). **b** KEGG enrichment analysis of up-regulated genes (*p* value-corrected < 0.05). **c** KEGG enrichment analysis of down-regulated genes (*p* value-uncorrected < 0.05). **d** KEGG enrichment analysis of up-regulated genes related to fatty acid metabolism (*p* value-corrected < 0.05). **e** KEGG enrichment analysis of down-regulated genes related to fatty acid metabolism (*p* value-corrected < 0.05)

The GO enrichment function of DEGs showed that the GO terms were mainly concentrated on the regulation of cytoskeleton, cell components and actin, and the functions related to protein and growth including photosynthesis pathway, light harvesting in photosystem I pathway, and regulation of protein polymerization pathway (Fig. 3a). In all up-regulated DEG sets, KEGG enrichment pathways (*p*-value corrected < 0.05) were mainly photosynthesis-antenna protein pathway, photosynthesis pathway, endocytosis pathway, phosphonate and phosphinate metabolism pathway, and protein processing in endoplasmic reticulum pathway (Fig. 3b). In all down-regulated DEG sets, no KEGG enrichment pathway was obtained by *p*-value corrected < 0.05; when* p*-value uncorrected < 0.05 was applied, the KEGG pathways obtained were mainly homologous recombination pathway, plant hormone signal transduction pathway, other glycan degradation pathway, MAPK signaling pathway-plant pathway, nucleotide excision repair pathway, SNARE interactions in vesicular transport pathway, starch and sucrose metabolism pathway, non-homologous end-joining pathway, ribosome pathway, and ubiquitin-mediated proteolysis pathway (Fig. 3c). To further explore the pathways related to fat and fatty acid metabolism, gene function queries were used to further screen DEGs directly related to fatty acid metabolism in the total DEG set, and KEGG enrichment analysis was performed at the *p*-value corrected < 0.05 level. As shown in Fig. 3 d and e, the enriched pathways related to the metabolism of fatty acids in *C. vulgaris* mainly included the fatty acid degradation pathway, fatty acid biosynthesis pathway, biosynthesis of unsaturated fatty acid pathway, and fatty acid association pathway.

### RT-qPCR for analysis of expression of related genes

#### Pathways related to photosynthesis

**Fig. 4:**
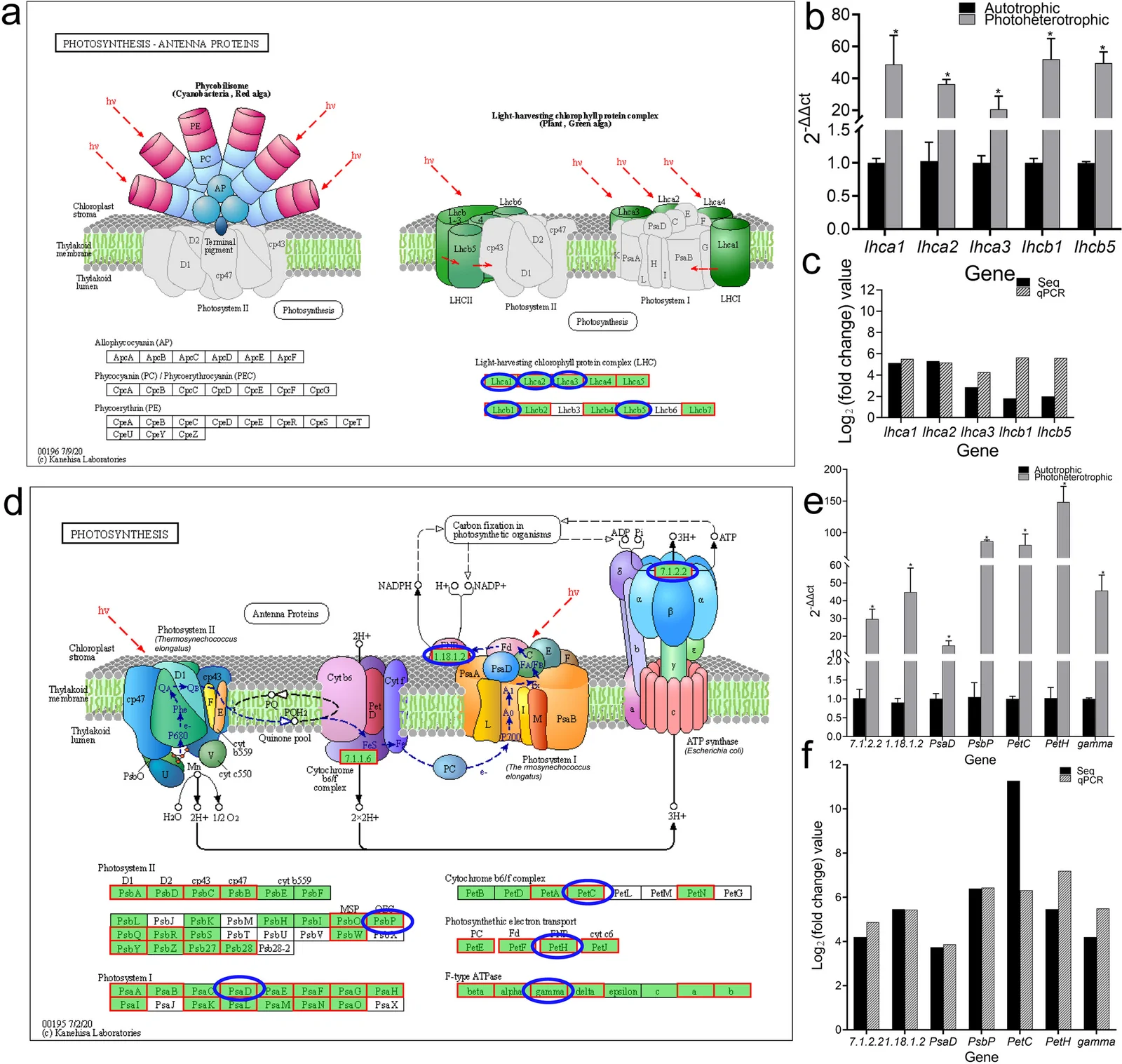
Pathways analysis related to photosynthesis. **a** Photosynthesis-antenna protein pathway (red color: up-regulated genes; blue color: high-expression genes) (Up-regulated gene set). **b** Photosynthesis pathway (up-regulated gene set). **c** Relative expression levels of differentially expressed genes between autotrophic and photoheterotrophic cultured *Chlorella vulgaris* (asterisk indicates a significant difference). **d** Comparison between RNA-Seq result and qPCR validation result. **e** Relative expression levels of differentially expressed genes between autotrophic and photoheterotrophic cultured *Chlorella vulgaris* (asterisk indicates a significant difference). **f** Comparison between RNA-Seq result and qPCR validation result

Among the up-regulated DEGs, KEGG was enriched in growth-related pathways including photosynthesis pathway and photosynthesis-antenna protein pathway (Fig. 4). For the DEGs in the photosynthesis-antenna protein pathway (Fig. 4a), qPCR analysis revealed that the expression of key DEGs in this pathway was significantly higher in the photoheterotrophic condition than that in the autotrophic condition (Fig. 4b). These PCR results were consistent with that in the transcriptome (Fig. 4c). Similarly, the expression of key DEGs in the photosynthesis-antenna protein pathway also was significantly higher in the photoheterotrophic condition than that in the autotrophic condition (Fig. 4d, e), which was consistent with that in the transcriptome (Fig. 4f).

#### Pathway analysis of protein synthesis and hydrolysis

**Fig. 5:**
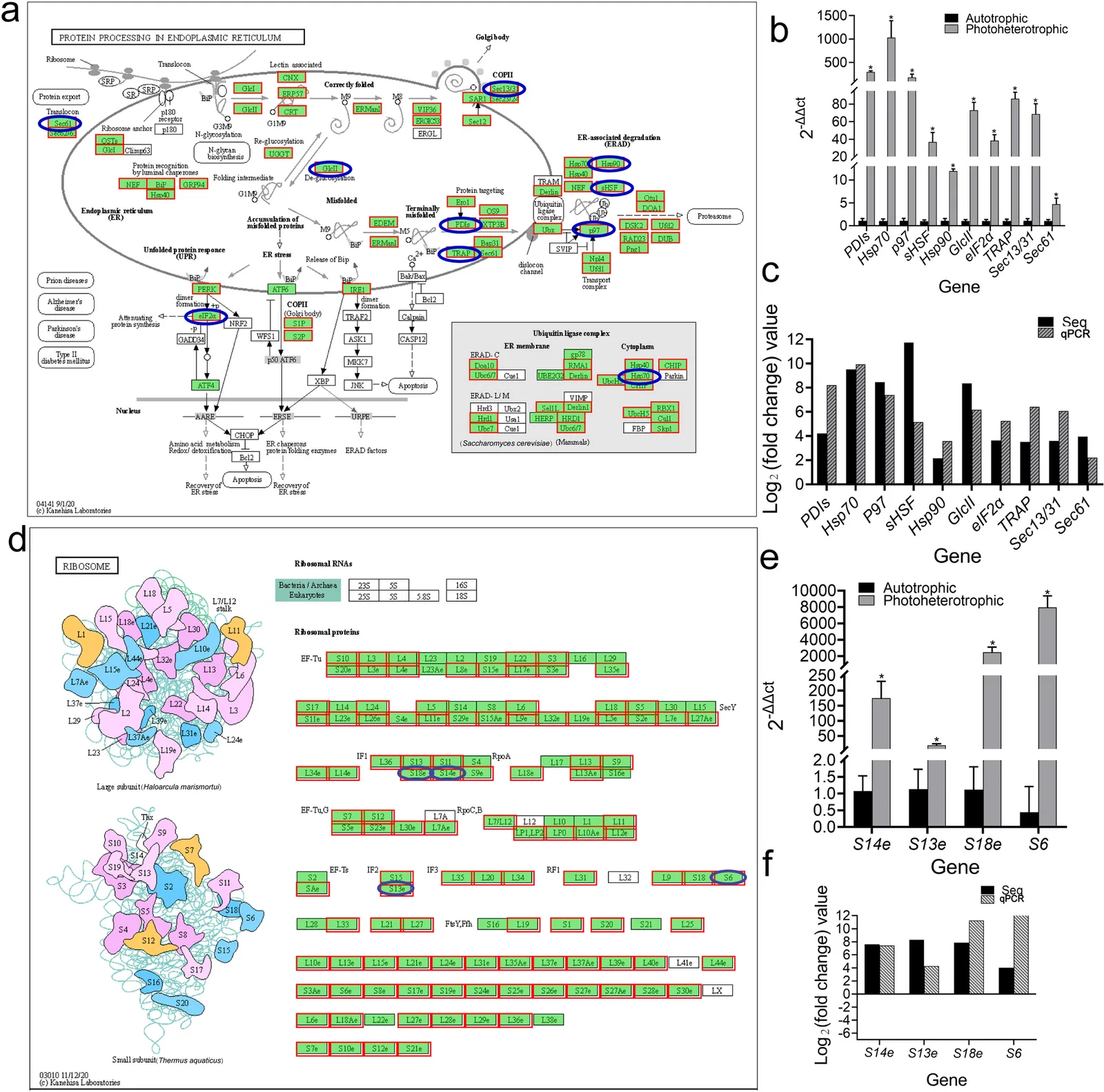
Pathway analysis related to protein synthesis and hydrolysis. **a** Protein processing in endoplasmic reticulum pathway (red color: up-regulated genes; blue color: high-expression genes) (up-regulated gene set). **b** Relative expression levels of differentially expressed genes between autotrophic and photoheterotrophic cultured *Chlorella vulgaris* (asterisk indicates a significant difference). **c** Comparison between RNA-Seq result and qPCR validation result. **d** Ribosome pathway (down-regulated gene set). **e** Relative expression levels of differentially expressed genes between autotrophic and photoheterotrophic cultured *Chlorella vulgaris* (asterisk indicates a significant difference). **f** Comparison between RNA-Seq result and qPCR validation result

Among the up-regulated DEGs, the KEGG pathway related to protein synthesis enriched was the protein processing in endoplasmic reticulum pathway (Fig. 5a). The relative expression of the key DEGs screened from this pathway in the photoheterotrophic condition is significantly higher than that in the autotrophic condition (Fig. 5 c and d). After correction by *p*-value-corrected < 0.05, it was found that no KEGG pathway was enriched from the down-regulated DEG set; however, judging by *p*-value-uncorrected < 0.05, one KEGG pathway related to protein synthesis and hydrolysis, the ribosome pathway was enriched (Fig. 5d). For key DEGs screened from this pathway, the relative expression of genes in the photoheterotrophic condition was significantly higher than that in autotrophic condition, which was consistent with that in the transcriptome (Fig. 5 e and f).

#### Pathway analysis of fatty acid metabolism

**Fig. 6:**
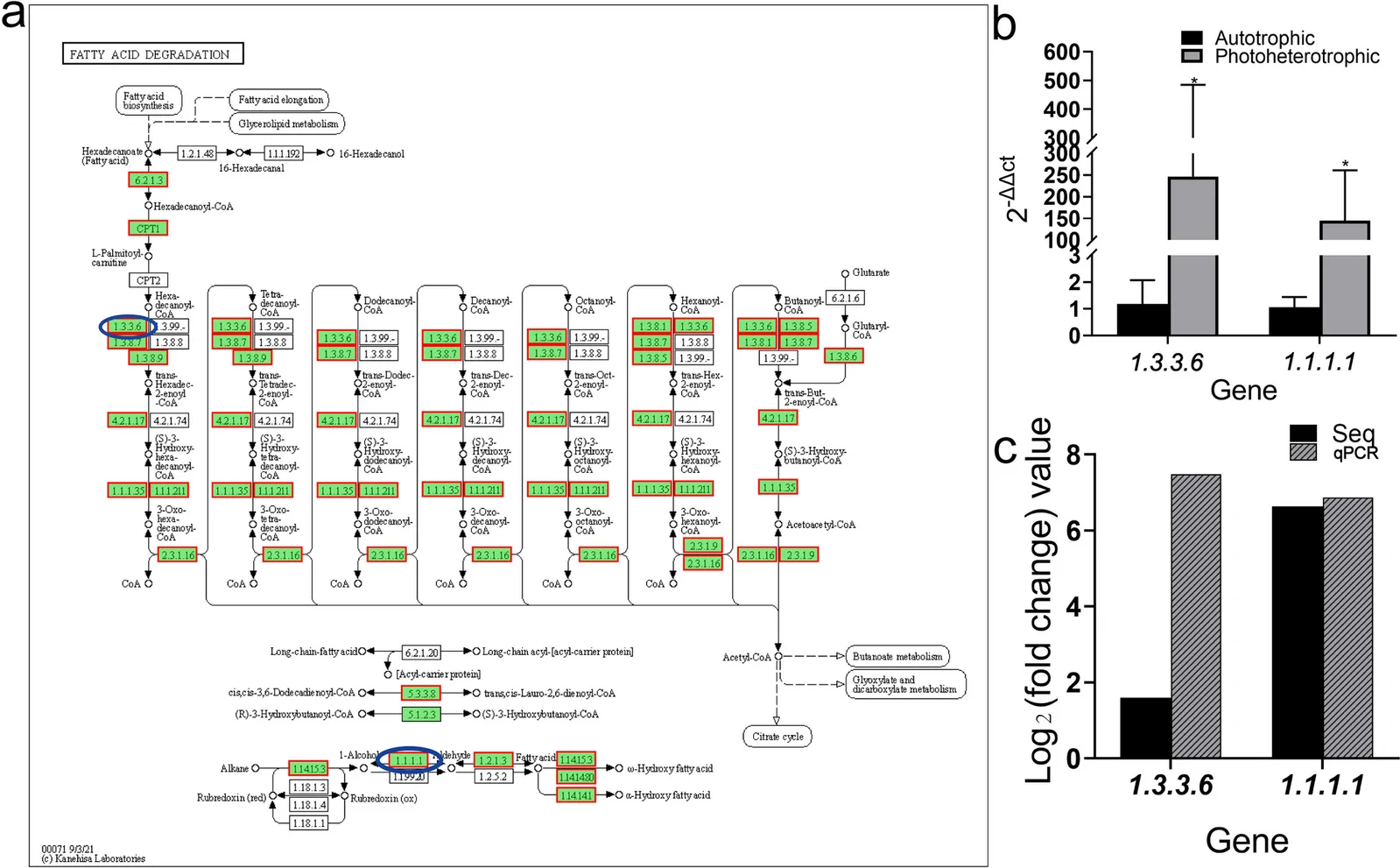
Pathway analysis related to fatty acid degradation. **a** Fatty acid degradation pathway (red color: up-regulated genes; blue color: high-expression genes) (up-regulated gene set). **b** Relative expression levels of differentially expressed genes between autotrophic and photoheterotrophic cultured *Chlorella vulgaris* (asterisk indicates a significant difference). **c** Comparison between RNA-Seq result and qPCR validation result

**Fig. 7:**
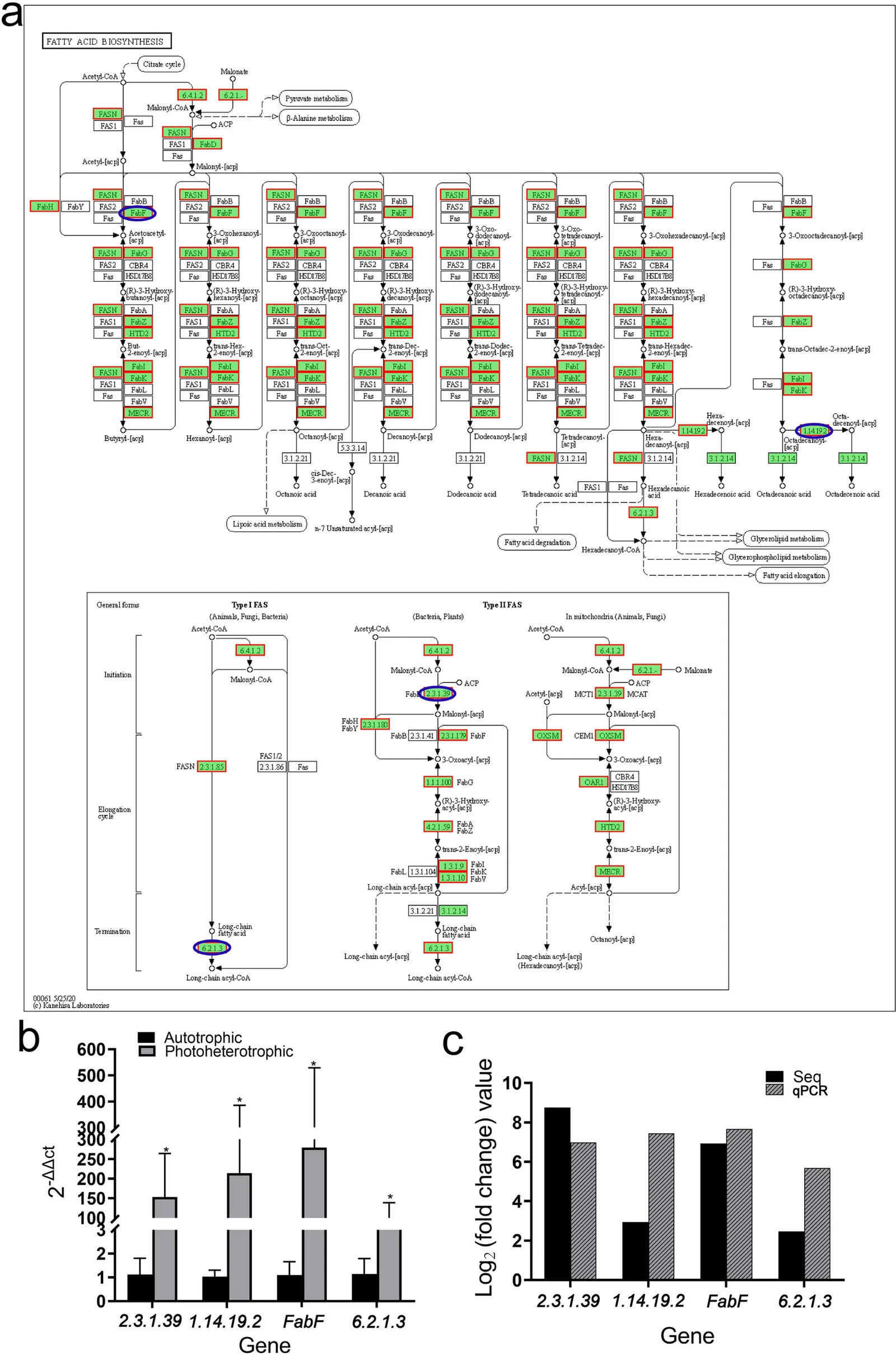
Pathway analysis related to fatty acid biosynthesis. **a** Fatty acid biosynthesis pathway (up-regulated gene set). **b** Relative expression levels of differentially expressed genes between autotrophic and photoheterotrophic cultured *Chlorella vulgaris* (asterisk indicates a significant difference). **c** Comparison between RNA-Seq result and qPCR validation result

After correction by *p*-value-corrected < 0.05, among all DEGs, the pathways associated with lipids were fatty acid degradation pathway (Fig. 6a) and fatty acid biosynthesis pathway (Fig. 7a), and the relative expression of key DEGs screened from these two pathways was significantly higher in the photoheterotrophic condition than in the autotrophic condition (Fig. 6b, c and Fig. 7 b and c).

## Discussion

### Effects of culture conditions on the growth and related genes

The current study showed that different culture conditions affected the growth of *Chlorella* cells. Generally speaking, the growth of the *Chlorella* under the 3 conditions showed the similar patterns with that obtained in a previous study, which all reached the peak value at the 6th or 7th day (Choong et al. 2020). In the natural environment, *Chlorella* can use either inorganic carbon sources (such as CO_2_) for phototrophy (Chisti 2007) or organic carbon sources (such as glucose) in the water for growing heterotrophically in the dark (Barta et al. 2021). Besides, it is reported that *Chlorella* can use both inorganic and organic carbon sources for synergy under the photoheterotrophic condition (Leong et al. 2022; Liang et al. 2009). Further, the growth rate and biomass of *Chlorella pyrenoidosa* under the photoheterotrophic condition were higher than those under the autotrophic condition (Li et al. 2022). Similarly, the growth rate and biomass of *C. vulgaris* under the photoheterotrophic condition were significantly higher than those under the heterotrophic condition and the autotrophic condition in this study, which may be the result of the synergistic effect of *Chlorella* using inorganic and organic carbon sources under light conditions (Leong et al. 2021; Li et al. 2022). Under the photoheterotrophic condition, *Chlorella* can simultaneously use CO_2_ in the air and endogenous CO_2_ produced by the heterotrophic pathway (Manhaeghe et al. 2020). Under the autotrophic condition, *Chlorella* will be affected by light intensity and CO_2_ supply. With the increase of cell density, the effect of photoinhibition will also increase. On the contrary, in the heterotrophic condition, organic carbon sources (such as glucose) are sufficient, so the biomass and growth rate of *Chlorella* under the heterotrophic condition should be significantly higher than that under the autotrophic condition (Kim et al. 2013; Liang et al. 2009; Zheng et al. 2012). However, in this study, from day 3, the biomass and growth rate of *Chlorella* under the autotrophic condition are higher than that under the heterotrophic condition. The reason for this phenomenon might be that *C. vulgaris* under the heterotrophic condition were polluted by other microorganisms, which competed for the nutrition of *Chlorella* and destroyed the pH of the culture medium (Rosli et al. 2019; Tian et al. 2020; Zhou et al. 2012).

The biggest difference between autotrophic condition and photoheterotrophic condition is whether they provide glucose. Therefore, these two cultivation conditions mainly affect *Chlorella* through photosynthesis. Through transcriptome analysis, it was found that under the photoheterotrophic condition, *Lhca1*, *Lhca2*, *Lhca3*, *Lhcb1*, and *Lhcb5* genes encoding light-harvesting chlorophyll protein complex (LHC) were up-regulated in the photosynthesis-antenna protein pathway, while *PsaD*, *PsbP*, *PetC*, *PetH*, and *gamma* genes encoding photosystem I and II, cytochrome b6/f complex, photosynthesis electron transport, and F-type ATPase were up-regulated in the photosynthetic pathway. Many microalgal organisms harvest light sources mainly through the light-harvesting chlorophyll protein complex (LHC); LHC protein is a membrane protein that is non-covalently attached to chlorophyll (Chl) and carotenoids (Car) (Büchel 2020). The roles of chlorophyll a (Chlor-a) and chlorophyll b (Chlor-b) in aquatic plants are very different, chlorophyll a plays the only role in blue-green algae, and both chlorophyll a and chlorophyll b play a role in green algae (Tanaka and Tanaka 2011). Two photosystems (PSI and PSII), cytochrome (Cyt) b6/f complex, and F-type ATP synthetase constitute the electron transport mechanism in photosynthesis (Yang et al. 2018). PsbP and PsbQ proteins are the external subunits of green algal photosystem II; PsbP plays an important role in the stable interaction of photosystem II and the ability of photosystem II to retain Ca^2+^ ions and Cl ions (Ifuku 2014). PsaD is one of the core protein complexes of the second photosystem of photosynthetic photoreaction, which uses light energy to produce high-energy carrier NADPH (Yang et al. 2018). Cytochrome b6/f complex mainly mediates the electron transfer of photosynthetic system I and II, in which nuclear *PetC* and *PetM* genes encode the Rieske iron-sulfur protein and a small peptide, respectively (Monde et al. 2000). Based on the abovementioned information, it can be inferred that the effect of photoheterotrophic condition on the growth of *Chlorella* might be mainly through the up-regulation of *Lhca1*, *Lhca2*, *Lhca3*, *Lhcb1*, *Lhcb5*, *PsaD*, *PsbP*, *PetC*, *PetH*, and *gamma* genes in chlorophyll a, chlorophyll b, and electron transport mechanism.

### Effects of culture conditions on lipid accumulation and fatty acid profile

The high lipid accumulation capacity of *Chlorella* is the key factor for the development of microalgal biodiesel. In this study, the lipid content of *Chlorella* under the photoheterotrophic condition was significantly higher than that under the heterotrophic condition. A possible reason for this phenomenon is that *Chlorella* can synthesize lipids through two ways: CO_2_ photosynthesis and oxidative phosphorylation of organic carbon sources (such as acetic acid or glucose) (Li et al. 2022). The lipid content of *Chlorella* under the autotrophic condition and heterotrophic condition in this study was different from the previously reported research results (Liang et al. 2009; Liu et al. 2011). In this study, the lipid content of *Chlorella* under the autotrophic condition was higher than that under the heterotrophic condition. The reason for this result may be that the poor growth at the harvesting time as *Chlorella* was polluted by other microorganisms under the heterotrophic condition. Specifically, SFA (saturated fatty acids) were the highest under the autotrophic condition, and MUFA and PUFA were the highest under the heterotrophic condition, while MUFA was the lowest under the autotrophic condition and PUFA was the lowest under the heterotrophic condition, which indicated that the photoheterotrophic condition would be a potential production method for high-quality algae rich in PUFA and MUFA, two important nutrients in human and animal nutrition. Under the heterotrophic and photoheterotrophic conditions with glucose as a carbon source, high oleic acid can better balance oxidation stability and low-temperature characteristics (Knothe 2009). The expression of all genes enriched in lipid degradation and fatty acid biosynthesis KEGG pathways was up-regulated under the photoheterotrophic condition, indicating that this culture mode can promote the lipid synthesis and metabolism in algal cells, while the expression of genes encoding enzymes related to fatty acid synthesis was up-regulated in algal cells under the photoheterotrophic status, indicating that the photoheterotrophic condition favors lipid synthesis in algal cells.

### Effects of culture conditions on protein production and related genes

From the results of this study, the protein content of *Chlorella* under the photoheterotrophic condition was 42.59%, which was higher than that under the heterotrophic condition and autotrophic condition (31.19% and 23.24%, respectively). This is consistent with the results of other researchers (Tian et al. 2020).

The protein will enter the endoplasmic reticulum for processing after the polypeptide chains are synthesized by ribosomes (Ciobanu et al. 2018; Ramakrishnan 2002). Key DEGs in the protein processing in the endoplasmic reticulum pathway and the ribosome pathway were up-regulated under the photoheterotrophic condition; it is speculated that the positive effect of the photoheterotrophic condition on protein synthesis of *Chlorella* is mainly realized through the protein processing in the endoplasmic reticulum pathway and the ribosome pathway. In the process of protein translation, the photoheterotrophic condition up-regulated the expression of *S13e*, *S14e*, *S18*e, and *S6* in ribosomes (Fig. 5e), while in protein processing in the endoplasmic reticulum pathway, the photoheterotrophic condition promoted the expression of *PDIs*, *Hsp70*, *Hsp90*, *p97*, *sHSF*, *G1cII*, *eIF2α*, *TRAP*, *Sec13/31*, and *Sec61* (Fig. 5c). IF1 (encoded by *S14e*, *S18e*), IF2 (encoded by *S13e*), and IF3 are three important initiation factors in protein translation; RF1 (S6) and RF2 are two “class I” release factors involved in recognizing stop codons (Ramakrishnan 2002); Sec61 is related to protein translocation and targeted delivery (Sitia and Braakman 2003); eIF2α is related to dimer formation and protein binding during protein synthesis (Sitia and Braakman 2003); the stress-inducible 70-kDa heat shock protein (Hsp70) belongs to the chaperone family, which mainly binds and releases polypeptides in the ATP-dependent cycle (Fewell et al. 2001); Hsp90 chaperones are abundant essential proteins that are associated with dimerization and preventing denatured proteins from aggregation in vitro (Fewell et al. 2001); and COPII (Sec13/31) is related to the formation of vesicles by protein packaging (Fewell et al. 2001). To sum up, as demonstrated by the results and literature data (Piasecka and Baier 2022), this study indicated that the photoheterotrophic condition mainly promotes the translocation, binding, de-glycosylation, dimer formation, protein targeted delivery, and vesicle formation of protein processing, thus to improve the protein content in the *C. vulgaris*.

To conclude, the effects of different culture conditions (autotrophic, heterotrophic, photoheterotrophic) on *Chlorella*’s growth and nutritional components was clarified in this current study, and potential mechanisms on the differences were analyzed at the transcriptome level through the key DEG identification and the KEGG pathway enrichment. Based on the results obtained in this study, the photoheterotrophic condition is considered as a suitable culture method for high-density, high-yield, short-cycle culture of *C. vulgaris*, a microalga with economic value, and can be applied to the biosynthesis of important microalgal active substances. This study can provide important ideas for the future use of algal resources to produce high-quality protein and fat sources for human food and fish feed. However, future studies regarding the effect of culture medium variation, temperature fluctuation, and light regime on the yield and quality of this *Chlorella* are desired for the high-efficiency production of *C. vulgaris* to meet the requirement of human food and aquatic feed. In addition, it is also necessary to further optimize the composition of photoheterotrophic culture media and culture conditions for the development of targeted culture of various functional *Chlorella*.

## Supplementary Information

Below is the link to the electronic supplementary material.

Supplementary file1 (PDF 213 KB)

## Data Availability

The datasets generated during and/or analyzed during the current study are available from the corresponding author on reasonable request. The transcriptome sequencing raw data had been deposited and the BioProject ID is PRJNA1007623.
