# Supplementary material for: Photoheterotroph improved the growth and nutrient levels of Chlorella vulgaris and the related molecular mechanism
Source: Appl Microbiol Biotechnol. 2024 Mar 20;108(1):269. doi: 10.1007/s00253-024-13090-w (PMC10954984; doi:10.1007/s00253-024-13090-w)
Supplement: Supplementary file 1 — Supplementary file1 (PDF 213 KB) [file 253_2024_13090_MOESM1_ESM.pdf]

Supplementary material for Applied Microbiology and Biotechnology

Photoheterotroph improved the growth and nutrient levels of *Chlorella vulgaris* and the related molecular mechanism

Xianmei Long[1]#, Cancan Zhang[1]#, Qian Yang[1], Xiaorui Zhang[1], Wangwang Chen[1], Xiaofang Zhu[2], Qing Xu[1], Qingsong Tan[1]*

[1]National Demonstration Center for Experimental Aquaculture Education, College of Fisheries, Huazhong Agricultural University, Wuhan, 430070, China; Key Laboratory of Freshwater Animal Breeding, Ministry of Agriculture, Wuhan 430070, China; Engineering Research Center of Green Development for Conventional Aquatic Biological Industry in the Yangtze River Economic Belt, Ministry of Education, Wuhan 430070, China

[2]Hubei Vocational College of Bio-technology, Wuhan 430070, China

The author unit of Cancan Zhang has been changed to School of Marine Sciences, Sun Yat-sen University, Zhuhai 519082, China

#These two authors contributed equally to this work.

***Corresponding author:**

Email: qstan@hotmail.com; tanqs2000@mail.hzau.edu.cn

Tel/Fax: 86-27-87282113

Table caption

Supplementary Table S1. BG11 medium formula

Supplementary Table S2. Preparation table of trace element solution A5

Supplementary Table S3. Key differential gene primers of *C. vulgaris*

Table S1. BG11 medium formula

Component	Content (mL/500mL)	Mother liquor concentration
$NaNO_3$	5	75 g/500 mL dH_2O
K_2HPO_4	5	2 g/500 mL dH_2O
$MgSO_4 \cdot 7H_2O$	5	3.75 g/500 mL dH_2O
$CaCl_2 \cdot 2H_2O$	5	1.8 g/500 mL dH_2O
Citric acid	5	0.3 g/500 mL dH_2O
Ferric ammonium citrate	5	0.3 g/500 mL dH_2O
EDTANa$_2$	5	0.05 g/500 mL dH_2O
Na_2CO_3	5	1.0 g/500 mL dH_2O
A5 (trace mental solution)	0.5	

Table S2. Preparation table of trace element solution A5

Component	Concentration
H_3BO_3	2.86 g/L dH_2O
$MnCl_2 \cdot 4H_2O$	1.86 g/L dH_2O
$ZnSO_4 \cdot 7H_2O$	0.22 g/L dH_2O
$Na_2MoO_4 \cdot 2H_2O$	0.39 g/L dH_2O
$CuSO_4 \cdot 5H_2O$	0.08 g/L dH_2O
$Co(NO_3)_2 \cdot 6H_2O$	0.05 g/L dH_2O

Table S3. Key differential gene primers of *C. vulgaris*

Gene name	Forward primer (5'-3')	Reverse primer (5'-3')	Gene ID
18S rRNA	CTGAGAAACGGCTAC-CACATC	CCCCACCCGAAATC-CAAC	X13688
PDIs	CGACATTGTTCGGTGGGTG	TCCACCAGCGCATACTTCG	PSC75600.1
Hsp70	CCAGCCAAGAAGAGCCAAAC	CACCTGCTGTCTCAATACCC	XP_003064239.1
Hsp90	CAGAAGAAGAAGCCCAACAACATCA	TCGGACTTGTCTGCCTCTGC	KAF6260424.1
p97	ATGCCAGAAGGTCGGTTAG	GGCAGCGACTGTAACCAAA	GAQ90153.1
sHSF	TACTTGGAGCAACTCTTACA	TCCACAGATTCAGGCAGTTT	GBG42469.1
G1cII	ACTACCACCGCTCTTCTCCTT	CTCGCCAGACTCAGACATCCA	GBG76832.1
eIF2α	TGCGCTCTATTGCGGAGAT	CGCTCCTCCTTCTCGCTGA	PRW56900.1
TRAP	TCAACACCGTGTCCGACTG	AAGGTGAAGGTGCGGAAGG	PSC69075.1
Sec13/31	TCTCCGATGCCAGCAACCAG	TGCCGCGTCTGCTCATTGTA	PSC72035.1
Sec61	AGATGCTGAACAACGGGTGG	CTTGAGCGCCTTGGTCTTGT	PRW61345.1
S13e	CTCGTTACTACCGTGCTTCT	TAGCATCCAACTTGCGAGAA	GAQ85311.1
S14e	TGGGTCGTAAAGTTGCTGAG	AATCGGTTGGGATAGGAGTG	KAF6262008.1
S18e	GAGGTATCCGTCACTTCTGG	CGATTTCAACTTCTGCCTTC	GBF94680.1
S6	TTTTACCTCTTGCCTATCC	TGTTGGTCCTTTCTCATTT	XP_022838335.1
7.1.2.2	GGAGGTGGACAAGGTGGAGC	GGGAGGAGGAGATGAGGGAC	PRW57448.1

1.18.1.2	GGCATCTGCTCCAACTTCC	TCCACAGACACCACCTTGC	XP_005848997.1
PsaD	CAGGCGCAGGTGGAGGAGTT	GTTCACGTTCTGGCCGATGC	PSC75399.1
PsbP	TCGGTTGCCTCCACCTTCG	CGTCGCCGTTGTCCTCATA	PRW60981.1
PetC	ATTGATGATGTCTGGTGCTG	GAGCTAAAGCGAGGGATAAA	QFB70700.1
PetH	GGGCATCTGCTCCAACTTCC	TGGAGGCGATGGAGTACAGG	XP_005848997.1
gamma	GGAGGTGGACAAGGTGGAGC	GGGAGGAGGAGATGAGGGAC	PRW57448.1
lhca1	GTGGCGGTGATTGTGCTTTC	TGCAAAGGTCACCACCTCGA	PRW45697.1
lhca2	TGCTTGGCGGCACCTACTGA	GGGACACGCCGTTGTTGATG	PSC72652.1
lhca3	TGCTGGCAATGTTCGGTTAC	GAGTGGGTCAAAGCCGAAGT	KAF6259926.1
lhcb1	AGCATGTTCGGCTTCTTCGT	GCTGTTGCCGAGGTAGTTCA	ABD37901.1
lhcb5	CTGGTGATTGCCATTGAGGT	ACGTGCTTGCTCCAGTTTGC	KAF6257400.1
1.3.3.6	GCCCGCTTCTTCTCGGTCTT	GGGTGTCGTGGTGCTTCTCC	PSC76022.1
2.3.1.39	CGCATCGCCAACTACCTGTG	CAGCGTCTTCAGCGTGGTCTC	XP_005850841.1
1.1.1.1	GGGCAAGCAACGATACGC	TCTCATCCAGGGCAGCAAAG	PSC69509.1
1.14.19.2	GGGCTTCATCTACACCTCCTTC	GCTTGCCGTCGTCCATCA	PSC67766.1
FabF	CAAGTTTGCGGCCCAGATC	TGTTGGTGATGGCGTAGGG	PSC74460.1
6.2.1.3	CGACAAGATCAAGCAGCACG	GCTGATGCGGACGGTGAT	PSC75938.1
